# 

**Published:** 1885-07

**Authors:** 


| | PAGE |
|---|---|
| ORIGINAL COMMUNICATIONS.—State Medicine. Dr. Frank S. Billings | 217 |
| Indigestion in the Horse. Charles J. Byrne, M.R.C.V.S. | 232 |
| Bacteria Culture. Dr. F. S. Billings | 240 |
| Public Abattoirs a Necessity to Public Health | 246 |
| Rudolf Virchow | 253 |
| The History of Tuberculosis. Dr. Albert Johne | 256 |
| EDITORIAL.—Spaying Bitches.—The Medical Diploma Craze.—Notes | 265 |
| CASE DEPARTMENT.—Autopsies at Central Park Menagerie.—Some Diseases of the Ourang-Outang | 269 |
| CORRESPONDENCE | 272 |
| PROCEEDINGS OF VETERINARY MEDICAL SOCIETIES.—N. Y. State Academy of Veterinary Science and Comparative Pathology.—Ontario Veterinary College.—The Chicago Veterinary College.—Annual Meeting of Massachusetts Veterinary Association | 276 |
| REVIEWS.—First Annual Report Bureau of Animal Industry.—Berlin as a Medical Centre.—Hay Fever.—Our Living World.—Twelfth Annual Report of the New Jersey Board of Agriculture.—Books and Pamphlets received | 285 |
| OBITUARY | 291 |
| PROGRESS OF VETERINARY SCIENCE | 291 |

